# Serum proteomic analysis uncovers novel serum biomarkers for depression

**DOI:** 10.3389/fpsyt.2024.1346151

**Published:** 2024-06-04

**Authors:** Aihong Guo, Bingju Wang, Jiangbo Ding, Lihong Zhao, Xiaofei Wang, Chen Huang, Bo Guo

**Affiliations:** ^1^ Department of Cell Biology and Genetics, School of Basic Medical Sciences, Xi’an Jiaotong University Health Science Center, Xi’an, China; ^2^ Department of Neurology, Xianyang Hospital of Yan’an University, Xianyang, China; ^3^ Department of Neurology, Rugao Hospital of Shenzhen Jingcheng Medical Group, Rugao, China; ^4^ Department of Dermatology, The Second Affiliated Hospital of Xi'an Jiaotong University, Xi’an, China; ^5^ Key Laboratory of Environment and Genes Related to Diseases, Ministry of Education, Xi’an Jiaotong University, Xi’an, China

**Keywords:** depression, serum, biomarker, mass spectrometry, early diagnosis

## Abstract

**Objective:**

The identification of depression primarily relies on the clinical symptoms and psychiatric evaluation of the patient, in the absence of objective and quantifiable biomarkers within clinical settings. This study aimed to explore potential serum biomarkers associated with depression.

**Methods:**

Serum samples from a training group comprising 48 depression patients and 48 healthy controls underwent proteomic analysis. Magnetic bead-based weak cation exchange (MB-WCX) and MALDI-TOF-MS were used in combination. To screen the differential peaks, ClinProTools software was employed. The proteins were identified using LC-MS/MS. ELISA was employed to confirm the expression of entire protein in the serum of the verification cohort, which encompassed 48 individuals who had been diagnosed with Depression and 48 healthy controls who were collected prospectively. Subsequently, logistic regression analysis was conducted to determine the diagnostic efficacy of the aforementioned predictors.

**Results:**

Five potential biomarker peaks indicating depression were identified in serum samples (peak 1, m/z: 1868.21; peak 2, m/z: 1062.35; peak 3, m/z: 1452.12; peak 4, m/z: 1208.72; peak 5, m/z: 1619.58). All of these peaks had higher expression in the pre-therapy group and were confirmed to be Tubulin beta chain (TUBB), Inter-alpha-trypsin inhibitor heavy chain H4 (ITIH4), Complement component 3 (C3), and Complement C4A precursor (C4A) by ELISA validation. Multivariate logistic regression analysis revealed that serum levels of TUBB, ITIH4, C3, and C4A were significant independent risk factors for the development of depression.

**Conclusion:**

Depression is a prevalent psychiatric condition. Timely detection is challenging, resulting in poor prognoses for patients. Our study on plasma proteomics for depression demonstrated that TUBB, ITIH4, C3, and C4A differentiate between depression patients and healthy controls. The proteins that were identified could potentially function as biomarkers for the diagnosis of depression. Pinpointing these biomarkers could enable early identification of depression, which would advance precise treatment.

## 1 Introduction

Depression is a prevalent and incapacitating psychiatric illness. It typically presents as profound and enduring mood depression. Certain cases exhibit noticeable anxiety and motor restlessness. Severe cases may feature hallucinations, delusions, suicidal tendencies, and other psychotic symptoms (1, 2). Depression is widespread, affecting almost one in five individuals at some point in their lives according to epidemiological data. In 2008, the World Health Organization ranked major depression as the third leading cause of burden of disease globally. They projected that the disease would become the first by 2030. Despite this projection, there continues to be an increase in depression’s incidence rate, particularly among younger generations (3, 4). In China, depression has been estimated to be the second leading cause of years lived with disability (5).

The diagnosis of depression is mainly based on the evaluation of patients’ clinical symptoms and signs and the evaluation of psychiatric examination scale include: Hamilton Depression Scale (HAMD) (6), Hamilton Anxiety Scale (HAMA) (7) etc. Patients experiencing low mood may exhibit a spectrum of affective states, ranging from sullenness to profound sadness, and may even manifest emotional numbing. Early diagnosis is difficult because patients often have insignificant early clinical symptoms (8). The mortality rate of depressed patients is about twice that of the general population, and their average life expectancy is 7–14 years lower (9). To date, there are no clinical blood indicators for diagnosing depression in the guidelines, and objective blood indicators for diagnosing and assessing the early onset of depression are urgently needed by clinicians.

In the process of exploring biomarkers for depression, body fluids such as blood, cerebrospinal fluid, urine, and saliva are the most practical specimens for patients to take samples. Of these, blood specimens are the most commonly used, and have many advantages in the search for biomarkers: First, blood specimens are relatively easy to obtain as biological specimens, and there are standard collection procedures in clinical testing that do not require invasive surgery to collect. The injury is smaller, reducing the discomfort and risk to the patient; Second, blood circulation circulates throughout the body’s vascular system and carries information about various biological molecules such as proteins, lipids, nucleic acids, and extracellular vesicles. It represents the physiological and pathological status of the entire body better and helps to have a comprehensive understanding of the disease; Third, blood specimens can be collected repeatedly, allowing for dynamic monitoring of the disease progression, which helps to understand the prognosis and treatment effect of the disease.

MS-based high-throughput proteomics represents the primary technique for large-scale protein characterization (10). Thanks to the swift progress in MS instruments and experimental methods, MS-based proteomics has now emerged as a trustworthy and indispensable tool for exploring biological processes at the protein level (11, 12). Our laboratory has previously employed MS to screen for biomarkers in Henoch-Schönlein purpura in Chinese children (13), colorectal cancer patients (14), pancreatic cancer patients (15), childhood autism spectrum disorder (16), and sophisticated postoperative complications of liver transplantation (17). This paper aims to investigate serum biomarkers of depression using MS-based proteomics.

**Figure 1:**
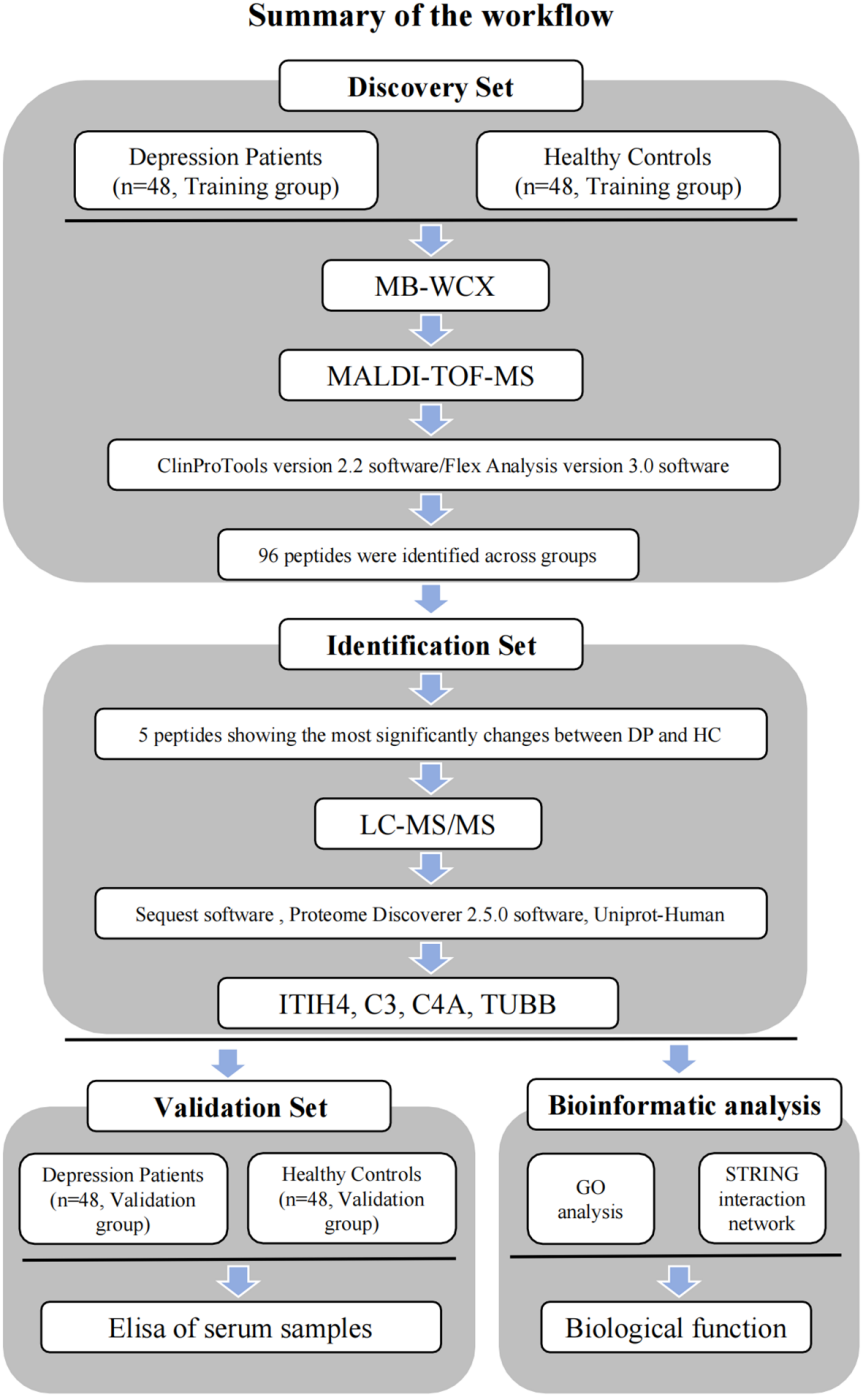
Workflow used for the discovery, identification, validation and bioinformatic analysis of potential biomarkers for depression.

MB-WCX purification was used in combination with MALDI-TOF-MS to conduct a proteomic analysis of samples from both depression patients and healthy controls. Comparisons of the abundance of generated serum proteomic profiles were performed with ClinProTools software. Peaks that were significantly differentially expressed between depression patients and healthy controls were considered potential diagnostic biomarkers and were subsequently sequenced and identified. Potential biomarkers were examined using bioinformatics and confirmed by ELISA (**Figure 1**). Subsequently, a logistic regression analysis was conducted to determine the diagnostic efficacy of the predictors.

## 2 Materials and methods

### 2.1 Patients and samples

This study received approval from the Ethics Committee of the Xianyang Hospital of Yan’an University (YDXY-KY-2020–016). Fasting serum samples were acquired from depression patients at the Xianyang Hospital of Yan’an University’s Department of Psychiatry from September 2020 to September 2022. Likewise, fasting serum samples from healthy controls were sourced from the Department of Physical Examination at Xianyang Hospital of Yan’an University during the same period. Informed consent was obtained from the patients and volunteers. The criteria for depression included patients aged 8 to 80 years who had a diagnosis of depression based on the Chinese Classification and Diagnostic Criteria of Mental Disorders (CCMD), the criteria and diagnosis of depression in the CCMD are reliable and agree well with ICD-10. In this study, the depressive patients included were all primary depressive patients who had not received any treatment before the onset of the illness and had no other relevant psychiatric comorbidities to the onset. Patients with tumors, hematological system diseases, severe immune system disease or heart, kidney, liver serious insufficiency, congenital, or hereditary diseases were excluded.

A total of 192 serum samples were collected from 96 depression patients, median (IQR) age 57(39.75, 65), 31.25% male, 68.75% female, and 96 healthy controls, median (IQR) age 59(46, 65.25), 35.42% male, 64.58% female. According to the 1:1 ratio, the 192 samples were randomly divided into the training group (48 depression patients, 48 healthy controls) and the verification group (48 depression patients, 48 healthy controls). The training group was used to screen the markers by mass spectrometry, and the validation group was used to validate the diagnostic efficacy of the candidate markers by ELISA. The baseline characteristics of the depression patients and healthy controls are shown in **Tables 1**, **2** and **Supplementary Table 1**.

**Table 1:** Baseline characteristics of depression patients and healthy controls (training group).

Characteristics	Depression(n=48)	Healthy control(n=48)	*P*-value
Onset age (years)	58 (43, 68.25)	56 (43.25, 64)	0.401
male	15(15.625%)	18(18.75%)	0.519
Serum AST U/L	17.5 (13, 23.25)	20 (14.75, 26)	0.357
Serum ALP U/L	84.5 (70.25, 106)	89 (72.25, 112.75)	0.437
Serum GGT U/L	22 (16, 37.5)	21 (13.75, 28.25)	0.559
Serum TBA μmol/L	4 (2.375, 6.05)	4.6 (2.05, 6.55)	0.857
Serum ALT U/L	13 (9.75, 18)	16 (11, 26)	0.173
Serum TP g/L	69.1 (65.375, 73.625)	71.4 (66.875, 76.5)	0.127
Serum ALB g/L	43.8 (41.65, 45.225)	45.4 (41.575, 48.45)	0.064
Serum GLO g/L	25.25 (23.4, 28.25)	25.95 (22.55, 28.45)	0.739
Serum TBIL μmol/L	11.05 (8.8, 15.475)	11.25 (8.4, 15.25)	0.832
Serum ADA U/L	7.246 ± 2.595	7.227 ± 2.504	0.971
Serum GLDH U/L	2.6 (1.675, 4.45)	2.5 (1.825, 4.3)	0.915
Serum CHE U/L	8052.7 ± 1333.5	8219.5 ± 1321.8	0.540

**Table 2:** Baseline characteristics of depression patients and healthy controls (verification group).

Characteristics	Depression(n=48)	Healthy control (n=48)	*P*-value
Onset age (years)	54.5 (38.5, 62)	52.5 (39, 64)	0.968
male	15(15.625%)	16(16.67%)	0.827
Serum AST U/L	19 (16, 25.25)	18 (15.75, 24.25)	0.520
Serum ALP U/L	81 (68.25, 101.25)	86 (68.75, 99.5)	0.714
Serum GGT U/L	19 (15, 26.75)	19.5 (16, 30.5)	0.486
Serum TBA μmol/L	4.05 (1.875, 6.025)	4.3 (2.5, 5.975)	0.626
Serum ALT U/L	17 (12, 22.5)	16 (11, 22)	0.402
Serum TP g/L	71.85 (66.9, 78.75)	71.3 (66.4, 74.6)	0.245
Serum ALB g/L	43.75 (41.425, 47.45)	43.9 (41.775, 45.65)	0.526
Serum GLO g/L	27.45 (22.75, 32.8)	26 (23.6, 29.05)	0.222
Serum TBIL μmol/L	12.21 ± 4.524	11.273 ± 4.146	0.293
Serum ADA U/L	6.95 (5.4, 8.65)	7.65 (5.725, 9.725)	0.385
Serum GLDH U/L	2.6 (1.775, 4.3)	2.85 (2.125, 4.9)	0.340
Serum CHE U/L	7882.2 ± 1809	8154.6 ± 1344.5	0.405

All serum samples were collected in 5 mL vacuum tubes without anticoagulant, then the samples were allowed to stand at 4 °C for 1 h, centrifuged at 3000 xg for 20 min at 4 °C and stored at -80 °C until use.

### 2.2 MS analysis

Serum samples were separated using MB-WCX (ClinProt purification reagent kits; Bruker Daltonics, Bremen, Germany). According to the standard protocol described by Bruker, the sample was bound to the magnetic beads. The magnetic beads were thoroughly mixed on a vortexing device for 1 minute.10 µL of binding buffer and 10 µL of MB-WCX beads were transferred to a standard thin-walled PCR tube and mixed by pipetting up and down.5 µL of serum was added to the solution and mixed intensively by pipetting up and down five times. The tube was left to incubate for 5 minutes. The tube was placed in the magnetic separator and the beads were collected on the wall of the tube for 1 minute. Carefully remove the supernatant with a pipette. Avoid contact of the pipette tips with the beads and take care not to remove the beads. Add 100 µL Wash Buffer to the tube. Collect the beads at the tube wall for 1 minute. Carefully remove the supernatant with a pipette. Repeat washing of the magnetic beads twice. Add 5 µL elution buffer and dissolve the beads from the tube wall by vigorous pipetting up and down 10 times. Collect the beads at the tube wall for 2 minutes. Transfer the clear supernatant to a fresh tube. Add 5 µL of stabilization buffer to the eluate and mix vigorously by pipetting up and down. The sample to be tested is obtained. Then apply 1 µL of sample to a target spot and allow to dry at room temperature (3–10 min). Apply 1 µL of matrix consisting of α-Cyano-4-hydroxycinnamic acid (HCCA) (3 mg/mL, in 50% acetonitrile/2% TFA) and allow to dry at room temperature. Each sample was spotted in triplicate to evaluate the reproducibility of the method.

### 2.3 ClinProTools analysis

Targets were immediately analyzed on a calibrated Autoflex III MALDI-TOF-MS (Bruker) using flexControl version 3.0 software (Bruker) and an optimized measurement protocol. Mass calibration was performed using a standard calibration mixture of peptides and proteins (mass range: 0.8–10 kDa). All assays were performed in a blinded fashion, including serum analyzes of different groups. Data analysis was performed using FlexAnalysis software (version 3.0; Bruker). Peptide pattern recognition was performed using ClinProTools version 2.2 (Bruker Daltonics), including spectral pretreatment, peak selection and peak calculation operations.

### 2.4 Peptide identification

The peptides eluted from the magnetic beads and were analyzed by nano-UPLC-ESI-MS/MS using a nano Aquity UPLC (Waters Corporation, Milford, USA) coupled to a Orbitrap Fusion mass spectrometer (Thermo Fisher Scientific, Bremen, Germany). Samples of 20μL (the sample was diluted by 2 times) were loaded on a C18 precolumn (Symmetry®C18, 5 μm, 180 μm × 20 mm, nanoAcquity™Column) at 15 μL/min in 5% acetonitrile (Sigma-Aldrich, St Louis, MO, USA), 0.05% trifluoroacetic acid (Sigma-Aldrich) for 3 min. The precolumn was switched online with the analytical column (Symmetry®C18, 3.5 μm, 75 μm × 150 mm, nanoAcquity™ Column) equilibrated in 95% solvent A (5% acetonitrile, 0.1% formic acid; Sigma-Aldrich) and 5% solvent B (95% acetonitrile, 1.2% formic acid). Peptides were eluted using a 5% to 80% gradient of solvent B over 60 min at a flow rate of 600 nL/min. The Orbitrap Fusion mass spectrometer was operated in the data-dependent mode to switch automatically between MS and MS/MS acquisition. Full-scan survey MS spectra with 2 microscans (m/z 200–2000) were acquired with the Orbitrap with a mass resolution of 100000 at m/z 400, followed by 10 sequential LC-MS/MS scans. Dynamic exclusion was used with 2 repeat counts, 10 s repeat duration and 60 s exclusion duration. For MS/MS, charge state 1 was rejected and precursor ions were activated using 25% normalized collision energy at the default activation q of 0.25. The mass spectra were searched against the human Uniprot database (https://www.uniprot.org/) using Proteome Discoverer software (Version 2.5.0); To reduce false positive identification results, a decoy database containing the reverse sequences was appended to the database. The parameters for the search were as follows: no enzyme, the variable modification was oxidation of methionine, peptide tolerance, 20 ppm, MS/MS tolerance, 1.0 Da.

### 2.5 Bioinformatic analysis

We used gene ontology (GO) enrichment analysis to analyze the identified proteins. We used the STRING database as a source to build an interaction network of potential biomarkers.

### 2.6 Validation using ELISA

All serum sample assays were blinded, and the standards for the ELISA kits and samples were run in triplicate. The concentrations of TUBB, ITIH4, C3 and C4A were quantified using the human TUBB ELISA kit (No. HB2558-Hu, Shanghai Hengyuan Biotech, China), the human ITIH4 ELISA kit (No. HB2554-Hu, Shanghai Hengyuan Biotech, China), the human C3 ELISA Kit (No. HB1872-Hu, Shanghai Hengyuan Biotech, China), the human C4A ELISA Kit (No. HB1508-Hu, Shanghai Hengyuan Biotech, China), according to the manufacturer’s instructions. The OD was measured at 450 nm.

### 2.7 Statistical analysis

Statistical analysis was performed using SPSS version 27.0 and GraphPad Prism version 8.0 (GraphPad Software, San Diego, CA, USA), with all data presented as mean ± standard deviation or median and interquartile range. Statistical significance was set at *p* < 0.05. t-tests, one-way ANOVA, Mann-Whitney U tests and Kruskal-Wallis H tests were used to compare quantitative data. Chi-square tests were used to compare categorical variables. Based on the univariate analysis, statistically significant variables were selected for logistic regression analysis. Predictive variables were identified and an ROC curve was plotted to determine the AUC value.

## 3 Results

### 3.1 Serum proteomic profiles of different groups

**Figure 2:**
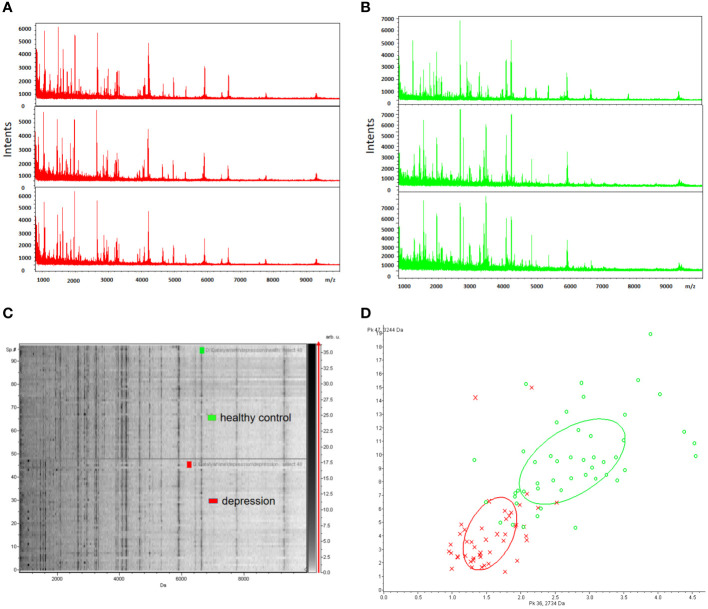
Reproducibility of mass spectra generated from individuals in different groups. **(A, B)** Representative mass spectra of healthy control (green), depression (red) serum samples (three spectra per sample) in the mass range from 0.8 to 10 kDa showing low variability between replicates of each sample. **(C)** Gel view of mass spectra of healthy control and depression serum samples in the mass range from 0.8 to 10 kDa showing low variability between replicates of each sample. **(D)** Depression (red) and healthy controls (green) in PCA with the two most differentiated peaks (m/z: 2734 Da, 3244 Da).

To ensure reproducibility and stability of the mass spectra, we analyzed all samples in triplicate and found highly reproducible peaks (**Figures 2A, B**). MB-WCX and MALDI-TOF-MS were used to compare the proteomic profiles of depression patients and healthy controls. Fractionation of serum samples showed that depression patients (red) and healthy controls (green) had proteomic profiles from 0.8 to 10 kDa (**Figures 2A, B**). The differentially expressed peaks between the two groups were detected in this mass range. According to the serum proteomics analysis, only a few overlapping areas were found between depression patients and healthy controls, indicating that the groups could be accurately distinguished (**Figures 2C, D**).

### 3.2 Peak selection and model testing

**Figure 3:**
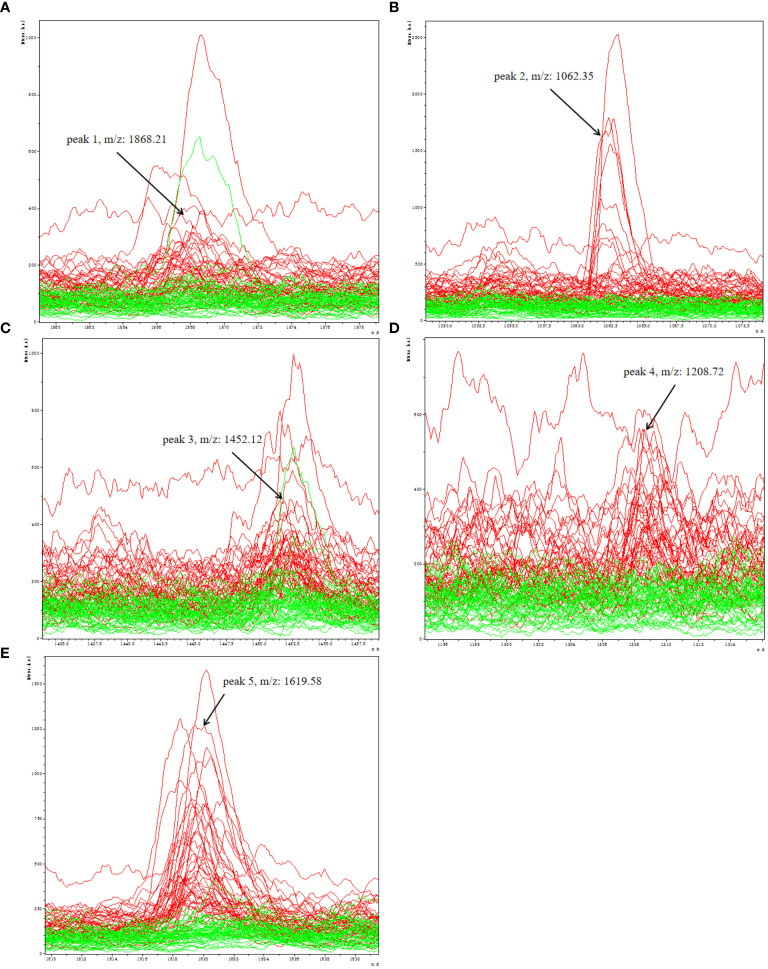
Representative spectra of five potential biomarker peaks in depression patients; comparison of the spectra of five peaks in depression patients (red) and healthy controls (green); **(A)** peak 1, m/z: 1868.21; **(B)** peak 2, m/z: 1062.35; **(C)** peak 3, m/z: 1452.12; **(D)** peak 4, m/z: 1208.72; **(E)** peak 5, m/z: 1619.58.

A total of 96 peaks, expressed as mean ± standard deviation, were identified in all groups analyzed. The information about all 96 peaks was added in **Supplementary Table 2**. The expressing levels of 96 peaks were showed by a heat map (**Supplementary Figure 1**). After obtaining 96 peaks, we further screened them based on fold change (FC) and significance level (*P*-value). The threshold standard was set as FC > 1.5 or FC < 0.67 and *p* < 0.05 (|log_2_FC| > 0.58 & *p* < 0.05). Subsequently, a volcano plot was generated, revealing that the depression group exhibited significant up-regulation of 5 peaks and down-regulation of 16 peaks(**Supplementary Figure 2**). Considering the significant time and financial costs associated with annotating all 21 candidate peptides that are significantly up- or down-regulated using LC-ESI-MS/MS, as well as the challenges of detecting low-abundance down-regulated peptides with low sensitivity and susceptibility to technical variability and measurement noise leading to false negative results, which can compromise diagnostic accuracy in clinical serum biomarker detection, we have selected a subset of upregulated peptides for further molecular identification annotation. Specifically, five peaks (peak 1, m/z: 1868.21; peak 2, m/z: 1062.35; peak 3, m/z: 1452.12; peak 4, m/z: 1208.72; peak 5, m/z:1619.58) were chosen for further analysis (**Figures 3A–E**).

### 3.3 Peak identification

All five peptide peaks (peak 1, m/z: 1868.21; peak 2, m/z: 1062.35; peak 3, m/z: 1452.12; peak 4, m/z: 1208.72; peak 5, m/z: 1619.58) that differed significantly between healthy controls and depression patients (**Table 3**) were identified using LC-ESI-MS/MS and the UniProt database. We identified proteins from the MS/MS spectra of these peptides, including TUBB, ITIH4, C3, C4A (**Table 4**).

**Table 3:** Mean levels of five differentially expressed proteins in controls and patients with depression.

Peak	Mass (Da) m/z	*P*-value	Depression (n=48)	Healthy Control (n=48)	Fold expression (depression/control)
1	1868.21	<0.000001	3.86 ± 2.27	2.53 ± 1.85	1.53↑
2	1062.35	<0.000001	9.09 ± 5.23	2.80 ± 0.60	3.25↑
3	1452.12	<0.000001	5.51 ± 1.79	3.51 ± 1.50	1.57↑
4	1208.72	<0.000001	4.66 ± 1.76	2.53 ± 0.60	1.84↑
5	1619.58	<0.000001	10.4 ± 5.85	3.66 ± 0.74	2.84↑

"↑" means up-regulation.

**Table 4:** Sequencing identification of five differentially expressed peaks between depression patients and healthy controls.

Peak	Mass (Da) m/z	Peptide Regions	Peptide sequence	UniProt ID	Identified protein
1	1868.21	363–379	K.MAVTFIGNSTAIQELFK.R	P07437	Tubulin beta chain (TUBB)
2	1062.35	489–498	G.SEMVVAGKLQ.D	Q14624	Inter-alpha-trypsin inhibitor heavy chain H4 (ITIH4)
3	1452.12	1308–1319	I.THRIHWESASLL.R	P01024	Complement C3 (C3)
4	1208.72	1342–1351	S.HALQLNNRQI.R	P0C0L4	Complement C4-A(C4A)
5	1619.58	63–78	R.AILVDLEPGTMDSVR.S	P07437	Tubulin beta chain (TUBB)

### 3.4 Full protein expression of identified peptides from different groups

**Figure 4:**
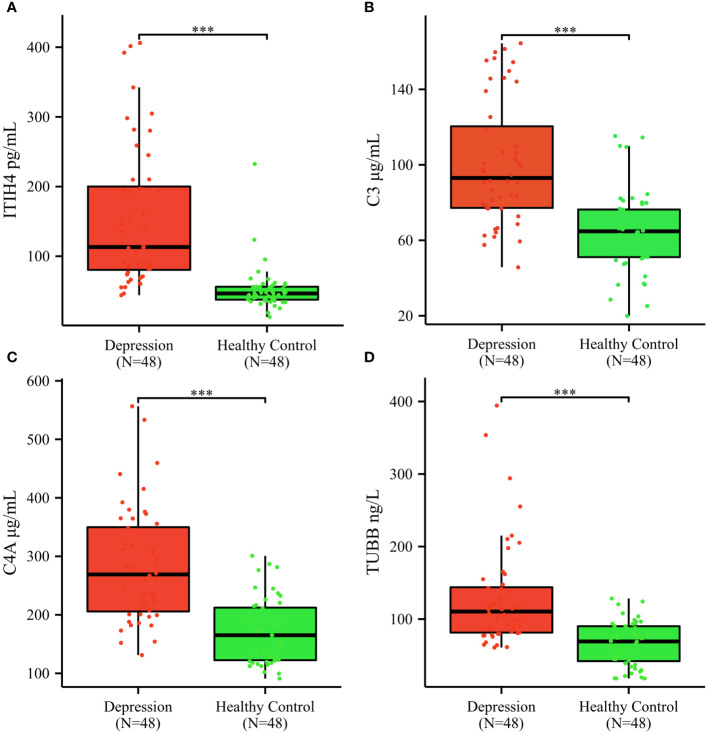
Serum ITIH4, C3, C4A, TUBB concentrations determined by ELISA in the validation cohort. **(A)** ITIH4; **(B) **C3; **(C)** C4A; **(D)** TUBB; The y-axis represents the protein concentration. The x-axis represents the validation groups. (*** indicates *p* < 0.001).

The mean serum concentrations of ITIH4, C3, C4A, TUBB were 154.77 ± 100.75 pg/mL, 100.70 ± 32.89 μg/mL, 283.20 ± 97.17 μg/mL, 129.78 ± 72. 80 ng/mL in depression patients; 52.11 ± 32.23 pg/mL, 65.03 ± 21.40 μg/mL, 171.50 ± 55.37μg/mL, 66.17 ± 30.48 ng/mL in healthy controls; (**Figures 4A–D**). The median serum concentrations of ITIH4 were 113.22 pg/mL, 46.57 pg/mL in depression patients and healthy controls, respectively, with a significant difference between the two groups (**Figure 4A**). The median serum concentrations of C3 were 93.03μg/mL, 64.77μg/mL in depression patients, healthy controls, respectively, with significant difference between the two groups (**Figure 4B**). The median serum concentrations of C4A were 269.05μg/mL, 165.11μg/mL in depression patients, healthy controls, respectively, with significant difference between the two groups (**Figure 4C**). The median serum concentrations of TUBB were 110.42 ng/mL and 69.20 ng/mL in depression patients and healthy controls, respectively, with a significant difference between the two groups (**Figure 4D**).

### 3.5 Forecast index ROC curve

**Figure 5:**
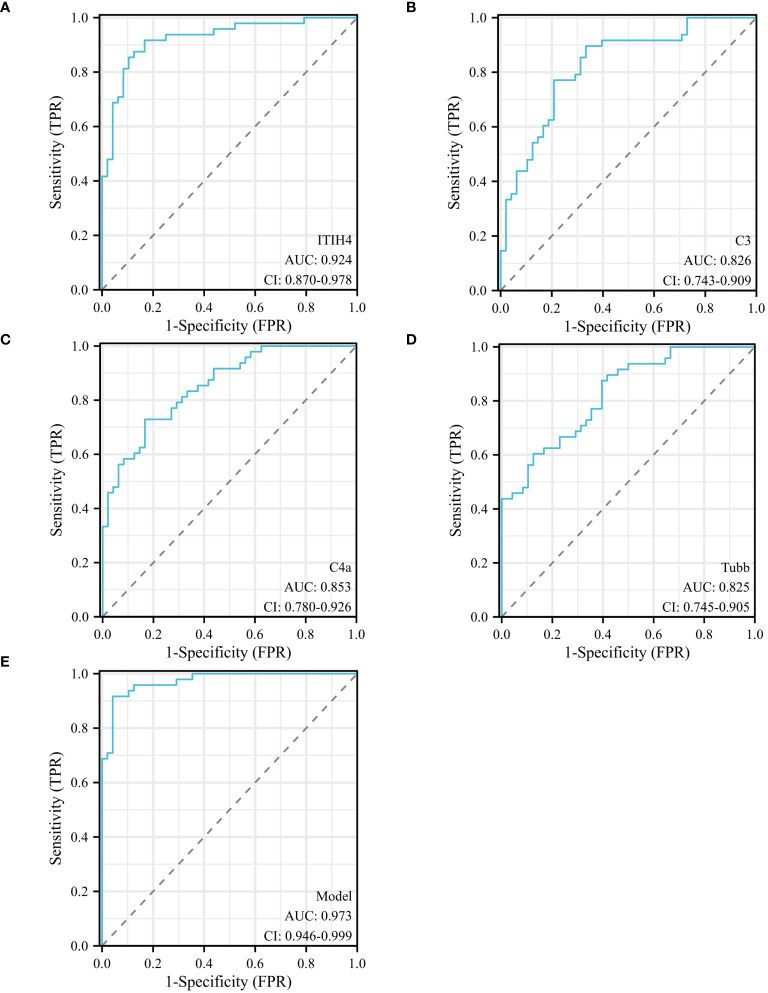
ROC curves of different predictors in different groups. **(A)** ROC curve analysis of serum ITIH4 levels to discriminate depression patients from healthy controls; **(B)** ROC curve analysis of serum C3 levels to discriminate depression patients from healthy controls; **(C)** ROC curve analysis of serum C4A levels to discriminate depression patients and healthy controls; **(D)** ROC curve analysis of serum TUBB levels to discriminate depression patients and healthy controls; **(E)** ROC curve analysis of combined serum ITIH4+C3+C4A+TUBB levels to discriminate depression patients and healthy controls; logit(Y) = 0.036×ITIH4 + 0.045×C3 + 0.023×C4A+0.035× TUBB -14.148.

Univariate analysis showed that ITIH4, C3, C4A and TUBB levels were significantly different between depression patients and healthy controls. ROC curves of ITIH4, C3, C4A and TUBB are shown in **Figures 5A–D**. Using serum levels of ITIH4, C3, C4A and TUBB, the AUC of the ITIH4 ROC curve was 0.924 (95% CI, 0.870–0. 978; *p* < 0.001, sensitivity 91.67%; specificity 83.33%; cutoff = 68.33; **Figure 5A**), the AUC of the C3 ROC curve was 0.826(95% CI, 0.743–0.909; *p* < 0.001, sensitivity 89. 58%; specificity 66.67%; cut-off value = 82.50; **Figure 5B**), the AUC of the C4A ROC curve was 0.853(95% CI, 0.780–0.926; *p* < 0.001, sensitivity 72.92%; specificity 83. 33%; cut-off value = 195.98; **Figure 5C**), the AUC of the TUBB ROC curve was 0.825 (95% CI, 0.745–0.905; *p* < 0.001, sensitivity 89.58%; specificity 58.33%; cut-off value = 99.50; **Figure 5D**). Multivariate logistic regression analysis identified serum ITIH4, C3, C4A and TUBB levels as independent risk factors for depression. Using the combination of serum ITIH4, C3, C4A and TUBB levels, the AUC of the ROC curve was 0.973 (95% CI, 0.946–0.999; *p* < 0.001, sensitivity 95.83%; specificity 91.67%; cut-off value = -0.536; **Figure 5E**).

### 3.6 Correlation analysis

Pearson’s analysis was used to evaluate the correlation between ITIH4, C3, C4A, TUBB, AST, ALP, GGT, TBA, ALT, TP, ALB, GLO, TBIL, ADA, GLDH, CHE; TUBB levels were significantly correlated with blood ITIH4 levels (r =0.435, *p* = 0.002) and blood GGT levels (r =0.431, *p* = 0.002). C3 levels correlated significantly with blood ADA levels (r = 0.320, *p* = 0.027).

### 3.7 Bioinformatic analysis of differentially expressed proteins

**Figure 6:**
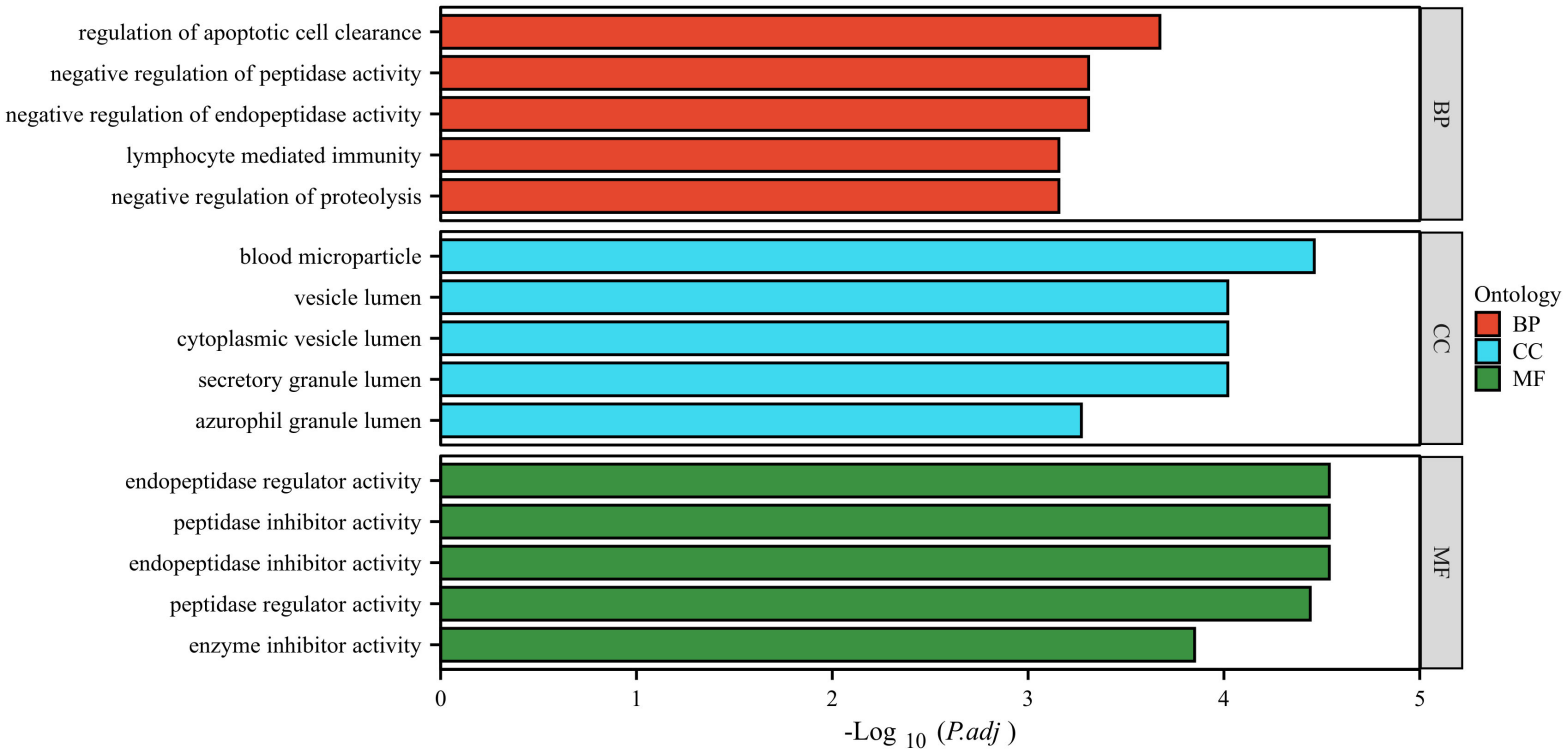
GO analysis of the four identified proteins. The ordinate represents the GO annotation classifications divided into three broad categories: biological process (BP), cellular component (CC) and molecular function (MF). The abscissa represents the -Log10 corrected *p*-value (*p*.adj).

The GO database analyzes gene classification annotation and biological function by biological process (BP), cellular component (CC) and molecular function (MF). The GO annotations for the four proteins were analyzed, and the results are shown in **Figure 6**. The four proteins were mainly involved in biological processes such as regulation of apoptotic cell clearance, negative regulation of peptidase activity, negative regulation of endopeptidase activity, lymphocyte-mediated immunity, negative regulation of proteolysis. The four proteins were mainly existed in cellular component such as blood microparticle, vesicle lumen, cytoplasmic vesicle lumen, secretory granule lumen, azurophil granule lumen. The four proteins were mainly involved in molecular function such as endopeptidase regulator activity, peptidase inhibitor activity, endopeptidase inhibitor activity, peptidase regulator activity, enzyme inhibitor activity.

**Figure 7:**
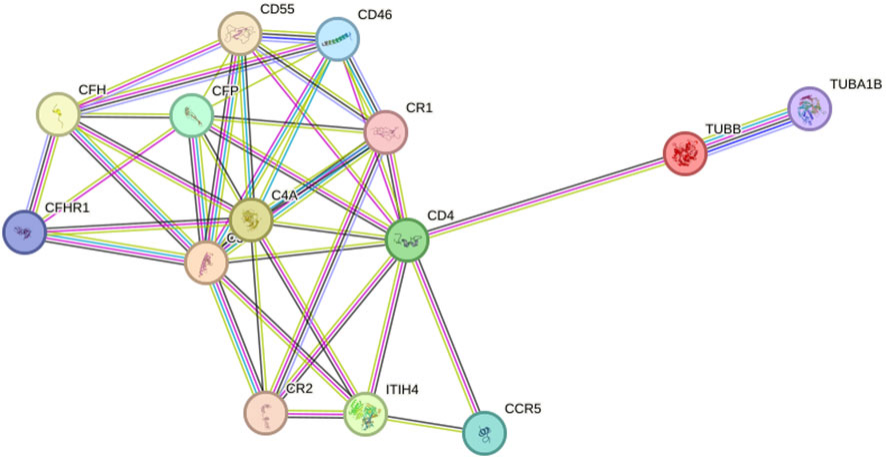
Interaction network between identified proteins and their function-related proteins (CCR5, CD4, CD46, CD55, CFH, CFHR1, CFP, CR1, CR2, TUBA1B) based on STRING prediction results.

To investigate the alteration of the protein interaction network in depression, we extracted networks using STRING software. A protein-protein interaction network was constructed using four seed proteins and their ten predicted functional partners in **Figure 7**. C-C chemokine receptor type 5 (CCR5), Cluster of differentiation 4 (CD4), Membrane cofactor protein (CD46), Complement decay-accelerating factor (CD55), Complement factor H (CFH), Complement factor H-related protein 1 (CFHR1); Properdin (CFP), Complement receptor type 1 (CR1), Complement receptor type 2 (CR2), Detyrosinated tubulin alpha-1B chain (TUBA1B).

## 4 Discussion

Currently, the clinical diagnosis of depression includes a medical history and psychiatric examination. Scale scoring often depends on the clinician’s diagnostic level, which is highly subjective. There is a lack of objective and quantifiable biological indicators in the clinical diagnosis of depression. Early diagnosis in depressed patients is difficult, especially for patients with early clinical manifestations who are not obvious lack of accurate psychological assessment. MALDI-TOF-MS can provide rapid, high-throughput protein analysis methods in proteomics research to help reveal the composition, structure and function of proteins, leading to in-depth understanding of biological processes and disease mechanisms. In this study, serum proteomic profiles of depression patients were generated using MB-WCX fractionation followed by MALDI-TOF-MS, which could accurately discriminate depression patients from healthy controls. Five peptides with the most significant changes in abundance between depression patients and were selected for further identification. Five peptides with upregulated expression were identified as peptide regions of TUBB (peak 1 m/z: 1868.21, peak 5 m/z: 1619.58), ITIH4 (peak 2 m/z: 1062.35), C3 (peak 3 m/z: 1452.12), C4A (peak 4 m/z: 1208.72). The results of the control group ELISA showed that serum levels of TUBB, ITIH4, C3 and C4A were significantly higher in patients with depression than in healthy controls.

TUBB is one of ten β-tubulin-coding genes in the human genome and is widely expressed in the developing central nervous system and skin (18). Tubulin is the major component of microtubules, a cylinder of laterally associated linear protofilaments composed of alpha- and beta-tubulin heterodimers, which are involved in neuronal proliferation, migration, differentiation, cargo transport along axons, synapse formation and many other functions (19). There are no other reports of TUBB in depression, so this study may indicate that TUBB is associated with depression.

ITIH4 is a liver-produced plasma protein that belongs to a family of proteins called the inter-α-inhibitor/ITIH family (20). Research on ITIH4 covers areas such as Sun et al (21) showed that ITIH4 is a key biomarker in the serum of patients with early gastric cancer and has potential as a high quality diagnostic marker for early gastric cancer. Huo et al (22). showed that serum ITIH4 may serve as an anti-inflammatory biomarker negatively associated with the degree of stenosis and the risk of major adverse cardiovascular events in patients with coronary artery disease. In addition, some studies have shown that elevated serum ITIH4 levels are associated with depression: Shi et al. (23) showed that elevated ITIH4 was consistently observed in both plasma and postmortem dorsolateral prefrontal cortex tissue of patients with major depressive disorder. Wang et al. (24) used iTRAQ technology and tandem mass spectrometry to show that serum ITIH4 levels were significantly elevated in depressed patients compared to healthy controls. The above results are consistent with our experimental findings that upregulated ITIH4 may be a potential biomarker for depression.

There is a growing body of evidence that the immune response has been implicated in the pathophysiology of depression (25–27).The complement system is an important component of immunity and one of its major effector mechanisms. The C3 and C4 play a key role in the complement cascade regulating inflammation and are particularly important in the activation of complement components. Luo et al (28) showed that the peripheral plasma concentration of C3 and C3a was significantly higher in the major depressive disorder group than in the healthy controls; Crider et al. (29) showed a significant increase in C3 expression in the prefrontal cortex of depressed suicide patients, and C4A is essential for the propagation of the classical complement pathway. Genetic deficiencies of C4A are the monogenic causative factors for the prototypic autoimmune disease systemic lupus erythematosus (30);Zhou et al. (31) showed that C4A deficiency is a risk factor for myositis, its subgroups and autoantibodies. To our knowledge, there are no reports on C4A expression in depression. The elevated C4A serum levels in depression patients in this study suggest that C4A may be a potential serum biomarker for depression.

Univariate and multivariate logistic regression analyzes of the clinical indicators of depression identified serum ITIH4, C3, C4A and TUBB levels as independent risk factors for depression. In addition, the combination of serum ITIH4, C3, C4A and TUBB levels increased sensitivity to 95.83% and specificity to 91.67%. The sensitivity and specificity of the new index were significantly higher than those of the previous single index.

GO and interaction network analyzes were performed to determine the biological significance of the four differentially expressed proteins. GO analysis showed that these proteins were involved in many biological processes, indicating the potentially complex pathogenesis of depression. STRING software showed that ITIH4, C3, C4A and TUBB were involved in the pathogenesis of depression through an unknown pathway.

In conclusion, our study preliminarily explored the potential serum peptide biomarkers for depression using proteomics-based MALDI-TOF MS; four proteins were identified as potential serum biomarkers from five significantly different polypeptides, and the corresponding total proteins were verified by ELISA. However, this study might have some limitations that merit consideration. First, the sample size included in this study is relatively small, we have not compared the expression of biomarkers between depression and other mental disorders; only possible candidate proteins involved are outlined. Second, the specific functions and mechanisms of action of these proteins require further investigation. Further studies of these serum biomarkers may provide greater insight into the molecular mechanisms involved in the etiology and development of depression. Third, the present study involved the screening and validation of four upregulated protein markers in individuals with depression. The downregulated biomarkers also hold significant biological significance and may potentially reflect mechanisms of disease suppression or other crucial physiological processes. In future investigations, we will explore the possibility of expanding the scope of our study to encompass in-depth examinations of down-regulated biomarkers associated with depression, aiming to provide a more comprehensive understanding and insights into disease mechanisms.

## Data availability statement

The datasets presented in this study can be found in online repositories. The names of the repository/repositories and accession number(s) can be found in the article/**Supplementary Material**.

## Ethics statement

The studies involving humans were approved by Ethics Committee of the Xianyang Hospital of Yan’an University (YDXY-KY-2020-016). The studies were conducted in accordance with the local legislation and institutional requirements. Written informed consent for participation in this study was provided by the participants’ legal guardians/next of kin.

## Author contributions

AG: Writing – review & editing, Writing – original draft. BW: Writing – original draft, Data curation. JD: Writing – original draft, Data curation. LZ: Writing – original draft, Data curation. XW: Writing – original draft, Data curation. CH: Writing – review & editing, Writing – original draft. BG: Writing – review & editing, Writing – original draft.
