# Supplementary material for: Serum proteomic analysis uncovers novel serum biomarkers for depression
Source: Front Psychiatry. 2024 Jun 4;15:1346151. doi: 10.3389/fpsyt.2024.1346151 (PMC11184055; doi:10.3389/fpsyt.2024.1346151)
Supplement: Supplementary file 1 [file DataSheet_1.docx]

Supplementary Material

# Supplementary Figures and Tables

## Supplementary Figures


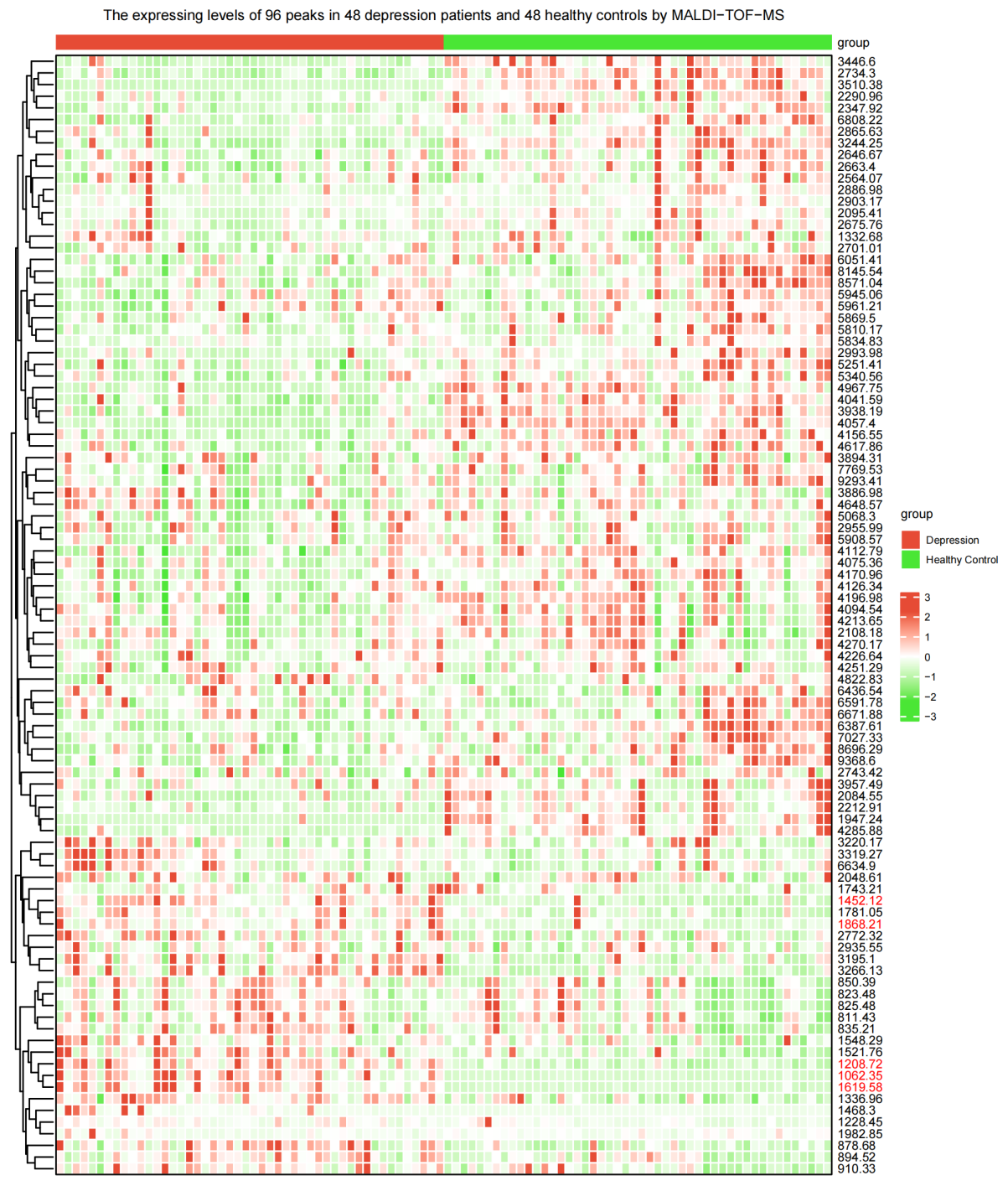


**Supplementary Figure 1.** The expressing levels of 96 peaks in 48 depression patients (red group) and 48 healthy controls (green group) by MALDI-TOF-MS. The horizontal coordinate represents the sample, the vertical coordinate represents the peaks, and the value of the heat map part has been converted by zscore.


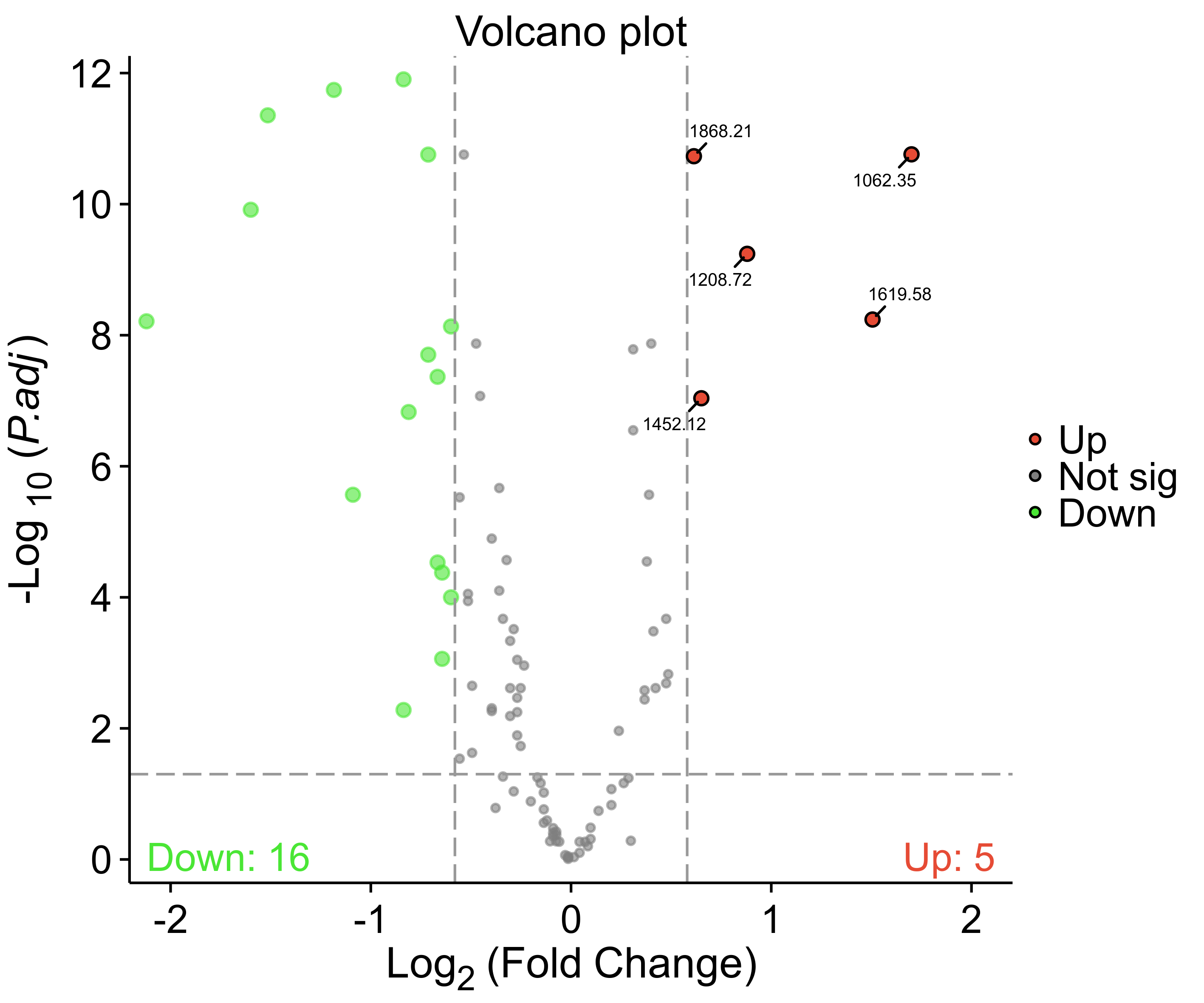


**Supplementary Figure 2.** Volcano plot. The differences of 96 peaks between depression and healthy controls are shown by fold change (depression vs healthy controls) and p value. Threshold: Log2FC (0.58), *p*.adj (0.05). Red dots indicate upregulated peaks in depression with *p* value<0.05 and Log2FC>0.58. Green dots indicate downregulated peaks in depression with *p* value<0.05 and Log2FC<-0.58.

## Supplementary Tables

**Supplementary Table 1.** Baseline characteristics of whole depression patients and healthy controls.

| Characteristics | Depression(n=96) | Healthy control(n=96) | *P*-value |
| --- | --- | --- | --- |
| Onset age (years) | 57 (39.75, 65) | 59 (46, 65.25) | 0.348 |
| male | 30 (15.6%) | 34 (17.7%) | 0.540 |
| Serum AST U/L | 18 (14, 24) | 18 (15, 26) | 0.772 |
| Serum ALP U/L | 83 (68.25, 102.75) | 88.5 (70, 105.25) | 0.404 |
| Serum GGT U/L | 19 (15, 34.25) | 20.5 (15, 29.25) | 0.904 |
| Serum TBA μmol/L | 4.05 (2.3, 6.025) | 4.4 (2.475, 6.325) | 0.624 |
| Serum ALT U/L | 15 (10, 20.5) | 16 (11, 22.25) | 0.618 |
| Serum TP g/L | 70.25 (66.375, 76.325) | 71.3 (66.4, 75.6) | 0.768 |
| Serum ALB g/L | 43.8 (41.5, 46.35) | 44.2 (41.675, 46.575) | 0.443 |
| Serum GLO g/L | 25.75 (22.875, 30.5) | 26 (23.2, 28.6) | 0.564 |
| Serum TBIL μmol/L | 11.6 (8.5, 15.975) | 11.3 (8.2, 14.35) | 0.382 |
| Serum ADA U/L | 6.95 (5.4, 8.725) | 7.4 (5.4, 9.15) | 0.522 |
| Serum GLDH U/L | 2.6 (1.7, 4.4) | 2.65 (1.9, 4.625) | 0.547 |
| Serum CHE U/L | 8158 (6764.5, 8958) | 8379 (7256, 9159.2) | 0.276 |

**Supplementary Table 2.** Mean levels of 96 differentially expressed proteins in controls and patients with depression

| Peak | Mass (Da) m/z | *P*-value | Depression(n=48) | Healthy Control(n=48) | Fold expression (depression/control) |
| --- | --- | --- | --- | --- | --- |
| 1 | 2734.3 | < 0.000001 | 1.72±0.43 | 3.06±0.86 | 0.56↓ |
| 2 | 3244.25 | < 0.000001 | 4.61±3.00 | 10.55±3.51 | 0.44↓ |
| 3 | 4057.4 | < 0.000001 | 2.99±1.56 | 8.56±3.85 | 0.35↓ |
| 4 | 1868.21 | < 0.000001 | 3.86±2.27 | 2.53±1.85 | 1.53↑ |
| 5 | 3938.19 | < 0.000001 | 1.82±0.54 | 2.96±0.78 | 0.61↓ |
| 6 | 4041.59 | < 0.000001 | 1.34±0.31 | 1.95±0.40 | 0.69↓ |
| 7 | 1062.35 | < 0.000001 | 9.09±5.23 | 2.8±0.60 | 3.25↑ |
| 8 | 3510.38 | < 0.000001 | 1.32±0.22 | 3.96±2.06 | 0.33↓ |
| 9 | 1947.24 | < 0.000001 | 2.82±2.27 | 12.46±8.69 | 0.23↓ |
| 10 | 1452.12 | < 0.000001 | 5.51±1.79 | 3.51±1.50 | 1.57↑ |
| 11 | 6387.61 | < 0.000001 | 0.66±0.13 | 1±0.31 | 0.66↓ |
| 12 | 1208.72 | < 0.000001 | 4.66±1.76 | 2.53±0.60 | 1.84↑ |
| 13 | 1619.58 | < 0.000001 | 10.4±5.85 | 3.66±0.74 | 2.84↑ |
| 14 | 1468.3 | < 0.000001 | 11.54±8.65 | 8.77±5.61 | 1.32↑ |
| 15 | 4156.55 | < 0.000001 | 1.63±0.33 | 2.26±0.55 | 0.72↓ |
| 16 | 3266.2 | < 0.000001 | 6.66±3.19 | 5.37±2.35 | 1.24↑ |
| 17 | 3446.6 | < 0.000001 | 1.59±0.42 | 2.59±0.93 | 0.61↓ |
| 18 | 2347.92 | < 0.000001 | 2.14±0.46 | 3.38±1.20 | 0.63↓ |
| 19 | 5251.41 | < 0.000001 | 0.88±0.21 | 1.2±0.28 | 0.73↓ |
| 20 | 4285.88 | < 0.000001 | 1.36±0.26 | 2.37±1.06 | 0.57↓ |
| 21 | 878.68 | < 0.000001 | 5.74±2.11 | 4.62±1.15 | 1.24↑ |
| 22 | 4112.79 | 0.00000215 | 1.53±0.38 | 1.96±0.40 | 0.78↓ |
| 23 | 4967.75 | 0.00000272 | 1.93±1.91 | 4.08±2.03 | 0.47↓ |
| 24 | 1336.96 | 0.00000272 | 3.82±0.87 | 2.92±0.78 | 1.31↑ |
| 25 | 6808.22 | 0.00000298 | 0.65±0.18 | 0.95±0.34 | 0.68↓ |
| 26 | 4617.86 | 0.0000127 | 1.18±0.29 | 1.56±0.45 | 0.76↓ |
| 27 | 7027.33 | 0.000027 | 0.77±0.14 | 0.96±0.24 | 0.80↓ |
| 28 | 1521.76 | 0.0000284 | 4.08±1.08 | 3.13±0.85 | 1.30↑ |
| 29 | 2084.55 | 0.0000294 | 2.82±1.41 | 4.46±1.96 | 0.63↓ |
| 30 | 5340.56 | 0.000042 | 2.65±1.35 | 4.13±1.77 | 0.64↓ |
| 31 | 8571.04 | 0.0000791 | 0.5±0.12 | 0.64±0.17 | 0.78↓ |
| 32 | 2212.91 | 0.0000886 | 2.26±0.54 | 3.23±1.39 | 0.70↓ |
| 33 | 2663.4 | 0.0001 | 21.57±12.03 | 32.53±12.55 | 0.66↓ |
| 34 | 2290.96 | 0.000114 | 2.14±0.49 | 3.04±1.33 | 0.70↓ |
| 35 | 3195.1 | 0.000213 | 4.3±1.69 | 3.09±1.03 | 1.39↑ |
| 36 | 4196.98 | 0.000213 | 2.56±0.77 | 3.23±0.81 | 0.79↓ |
| 37 | 4170.96 | 0.000306 | 1.82±0.43 | 2.21±0.50 | 0.82↓ |
| 38 | 2772.32 | 0.00033 | 3.1±1.10 | 2.33±0.74 | 1.33↑ |
| 39 | 9368.6 | 0.000462 | 0.75±0.17 | 0.93±0.27 | 0.81↓ |
| 40 | 2993.98 | 0.000869 | 2.58±1.94 | 4.04±1.91 | 0.64↓ |
| 41 | 2646.67 | 0.000899 | 2.4±0.46 | 2.88±0.77 | 0.83↓ |
| 42 | 6051.41 | 0.0011 | 0.82±0.16 | 0.96±0.21 | 0.85↓ |
| 43 | 3319.27 | 0.00149 | 5.14±2.51 | 3.68±1.27 | 1.40↑ |
| 44 | 894.52 | 0.00205 | 5.86±2.77 | 4.22±1.78 | 1.39↑ |
| 45 | 2675.76 | 0.00224 | 3.19±1.61 | 4.5±2.14 | 0.71↓ |
| 46 | 1548.29 | 0.00243 | 7.58±2.99 | 5.67±2.59 | 1.34↑ |
| 47 | 5810.17 | 0.00243 | 0.89±0.21 | 1.1±0.37 | 0.81↓ |
| 48 | 8696.29 | 0.00243 | 0.58±0.14 | 0.69±0.16 | 0.84↓ |
| 49 | 1781.05 | 0.00263 | 3.28±1.03 | 2.54±1.17 | 1.29↑ |
| 50 | 8145.54 | 0.00341 | 0.57±0.13 | 0.69±0.22 | 0.83↓ |
| 51 | 910.33 | 0.00362 | 5.46±2.06 | 4.23±1.67 | 1.29↑ |
| 52 | 4213.65 | 0.00494 | 17.52±6.94 | 23.16±10.58 | 0.76↓ |
| 53 | 2865.63 | 0.00524 | 4.24±4.38 | 7.57±6.13 | 0.56↓ |
| 54 | 9293.41 | 0.00543 | 1.9±0.80 | 2.5±1.11 | 0.76↓ |
| 55 | 4075.36 | 0.00565 | 1.96±0.50 | 2.37±0.80 | 0.83↓ |
| 56 | 7769.53 | 0.00648 | 1.71±0.61 | 2.1±0.65 | 0.81↓ |
| 57 | 835.21 | 0.0109 | 7.05±1.99 | 5.96±1.85 | 1.18↑ |
| 58 | 5869.5 | 0.0128 | 1.68±0.57 | 2.02±0.67 | 0.83↓ |
| 59 | 4094.54 | 0.0186 | 5.98±1.94 | 7.13±2.41 | 0.84↓ |
| 60 | 2095.41 | 0.0236 | 3.31±1.91 | 4.63±3.16 | 0.71↓ |
| 61 | 2886.98 | 0.029 | 2.73±2.27 | 4.02±2.97 | 0.68↓ |
| 62 | 5834.83 | 0.0544 | 0.84±0.21 | 1.07±0.72 | 0.79↓ |
| 63 | 5945.06 | 0.056 | 1.08±0.28 | 1.21±0.33 | 0.89↓ |
| 64 | 6634.9 | 0.0572 | 4.12±1.94 | 3.38±1.45 | 1.22↑ |
| 65 | 825.48 | 0.0684 | 11.69±4.71 | 9.71±4.86 | 1.20↑ |
| 66 | 4126.34 | 0.0684 | 1.84±0.44 | 2.04±0.55 | 0.90↓ |
| 67 | 850.39 | 0.0845 | 7.21±2.06 | 6.29±2.64 | 1.15↑ |
| 68 | 5908.57 | 0.0915 | 7.21±3.52 | 8.79±4.73 | 0.82↓ |
| 69 | 2743.42 | 0.0955 | 2.57±0.62 | 2.81±0.62 | 0.91↓ |
| 70 | 3957.49 | 0.13 | 3.67±1.27 | 4.21±1.80 | 0.87↓ |
| 71 | 823.48 | 0.148 | 9.05±3.42 | 7.89±3.56 | 1.15↑ |
| 72 | 2903.17 | 0.164 | 2.08±0.97 | 2.69±2.55 | 0.77↓ |
| 73 | 5068.3 | 0.172 | 1.25±0.45 | 1.38±0.41 | 0.91↓ |
| 74 | 4822.83 | 0.181 | 1.36±0.48 | 1.24±0.29 | 1.10↑ |
| 75 | 2701.01 | 0.254 | 2.3±0.88 | 2.51±0.64 | 0.92↓ |
| 76 | 2108.18 | 0.276 | 6.23±2.09 | 6.86±2.84 | 0.91↓ |
| 77 | 3220.17 | 0.328 | 2.62±0.76 | 2.45±0.70 | 1.07↑ |
| 78 | 2564.07 | 0.333 | 2.38±0.74 | 2.54±0.62 | 0.94↓ |
| 79 | 6671.88 | 0.377 | 1.03±0.23 | 1.08±0.25 | 0.95↓ |
| 80 | 3894.31 | 0.393 | 2.56±0.72 | 2.71±0.73 | 0.94↓ |
| 81 | 6591.78 | 0.421 | 0.96±0.23 | 1.01±0.31 | 0.95↓ |
| 82 | 4270.17 | 0.442 | 2.42±0.71 | 2.57±0.95 | 0.94↓ |
| 83 | 811.43 | 0.488 | 15.13±6.27 | 14.09±6.38 | 1.07↑ |
| 84 | 1982.85 | 0.519 | 3.31±5.54 | 2.7±0.68 | 1.23↑ |
| 85 | 2955.99 | 0.528 | 6.25±3.11 | 6.69±2.72 | 0.93↓ |
| 86 | 4648.57 | 0.528 | 4.76±2.05 | 5.03±1.69 | 0.95↓ |
| 87 | 5961.21 | 0.535 | 0.99±0.32 | 1.03±0.29 | 0.96↓ |
| 88 | 2048.61 | 0.537 | 3.11±0.77 | 3.01±0.72 | 1.03↑ |
| 89 | 2935.55 | 0.538 | 3.24±1.07 | 3.1±0.95 | 1.05↑ |
| 90 | 1743.21 | 0.628 | 4.46±1.96 | 4.2±2.73 | 1.06↑ |
| 91 | 4226.64 | 0.79 | 2.83±1.49 | 2.75±0.92 | 1.03↑ |
| 92 | 3886.98 | 0.86 | 3.29±1.28 | 3.35±1.02 | 0.98↓ |
| 93 | 1332.68 | 0.897 | 4.15±1.14 | 4.19±1.28 | 0.99↓ |
| 94 | 6436.54 | 0.921 | 1.79±0.60 | 1.78±0.59 | 1.01↑ |
| 95 | 4251.29 | 0.933 | 2.07±0.60 | 2.08±0.55 | 0.99↓ |
| 96 | 1228.45 | 0.978 | 3.76±1.11 | 3.78±5.09 | 0.99↓ |
